# Salience attribution and its relationship to cannabis-induced psychotic symptoms

**DOI:** 10.1017/S0033291716002051

**Published:** 2016-09-15

**Authors:** M. A. P. Bloomfield, E. Mouchlianitis, C. J. A. Morgan, T. P. Freeman, H. V. Curran, J. P. Roiser, O. D. Howes

**Affiliations:** 1 Psychiatric Imaging Group, MRC Clinical Sciences Centre, Institute of Clinical Sciences, Hammersmith Hospital, Imperial College London, Du Cane Road, London W12 0NN, UK; 2 Department of Psychosis Studies, Institute of Psychiatry, Psychology & Neuroscience, King's College London, De Crespigny Park, London SE5 8AF, UK; 3 Division of Psychiatry, University College London, 6th Floor Maple House, 149 Tottenham Court Road, London W1T 7NF, UK; 4 Clinical Psychopharmacology Unit, Research Department of Clinical, Educational and Health Psychology, University College London, 4th Floor, 1–19 Torrington Place, London WC1E 7HB, UK; 5 Washington Singer Laboratories, Department of Psychology, University of Exeter, Exeter EX4 4QG, UK; 6 Institute of Cognitive Neuroscience, University College London, 17 Queen Square, London WC1N 3AR, UK

**Keywords:** Addiction, cannabis, dopamine, psychosis, salience

## Abstract

**Background:**

Cannabis is a widely used drug associated with increased risk for psychosis. The dopamine hypothesis of psychosis postulates that altered salience processing leads to psychosis. We therefore tested the hypothesis that cannabis users exhibit aberrant salience and explored the relationship between aberrant salience and dopamine synthesis capacity.

**Method:**

We tested 17 cannabis users and 17 age- and sex-matched non-user controls using the Salience Attribution Test, a probabilistic reward-learning task. Within users, cannabis-induced psychotic symptoms were measured with the Psychotomimetic States Inventory. Dopamine synthesis capacity, indexed as the influx rate constant *K*_*i*_^*cer*^, was measured in 10 users and six controls with 3,4-dihydroxy-6-[^18^F]fluoro-l-phenylalanine positron emission tomography.

**Results:**

There was no significant difference in aberrant salience between the groups [*F*_1,32_ = 1.12, *p* = 0.30 (implicit); *F*_1,32_ = 1.09, *p* = 0.30 (explicit)]. Within users there was a significant positive relationship between cannabis-induced psychotic symptom severity and explicit aberrant salience scores (*r* = 0.61, *p* = 0.04) and there was a significant association between cannabis dependency/abuse status and high implicit aberrant salience scores (*F*_1,15_ = 5.8, *p* = 0.03). Within controls, implicit aberrant salience was inversely correlated with whole striatal dopamine synthesis capacity (*r* = −0.91, *p* = 0.01), whereas this relationship was non-significant within users (difference between correlations: *Z* = −2.05, *p* = 0.04).

**Conclusions:**

Aberrant salience is positively associated with cannabis-induced psychotic symptom severity, but is not seen in cannabis users overall. This is consistent with the hypothesis that the link between cannabis use and psychosis involves alterations in salience processing. Longitudinal studies are needed to determine whether these cognitive abnormalities are pre-existing or caused by long-term cannabis use.

## Introduction

Cannabis is a widely used drug (United Nations Office on Drugs and Crime, 2010) and cannabis may disrupt reward-based learning (Mendelson *et al.* 1976; Cherek *et al.* 2002; Lane & Cherek, 2002; Lane *et al.* 2005). The main psychoactive substance in cannabis is ∆^9^-tetrahydrocannabinol (THC) (Wachtel *et al.* 2002), an endocannbinoid CB_1_ receptor partial agonist (Felder *et al.* 1992; Sim *et al.* 1996; Petitet *et al.* 1998; Shen & Thayer, 1999; Breivogel & Childers, 2000; Govaerts *et al.* 2004; Kelley & Thayer, 2004; Paronis *et al.* 2012). Human and animal research indicates that THC can disrupt reward-based behaviour (Stiglick & Kalant, 1983; Foltin *et al.* 1989; Kamien *et al.* 1994; Lane & Cherek, 2002; Lane *et al.* 2004). The mesolimbic dopamine system mediates reward-based learning (Berridge & Robinson, 1998), which in turn is modulated by the endocannabinoid system (Fernandez-Ruiz *et al.* 2010; Melis & Pistis, 2012; Melis *et al.* 2012).

THC has complex effects on the dopamine system: studies in rodents indicate that acute administration increases dopaminergic neuron firing rates (French, 1997); whilst chronic administration reduces presynaptic dopaminergic function (Ginovart *et al.* 2012). In humans, acute THC administration has been reported to increase dopamine release in two out of four studies (Bossong *et al.* 2009; Stokes *et al.* 2009; Barkus *et al.* 2011; Kuepper *et al.* 2013), whilst chronic cannabis use is associated with reductions in dopaminergic function (Urban *et al.* 2012; Albrecht *et al.* 2013; Bloomfield *et al.* 2014*a*, *b*; Volkow *et al.* 2014). There is also evidence that long-term cannabis use is associated with attenuated striatal reward processing (van Hell *et al.* 2010). These studies provide converging evidence that cannabis use disrupts reward-based learning by changes to the dopaminergic system.

Cannabis users are dose-dependently at increased risk of schizophrenia (Murray *et al.* in press). Psychosis has been proposed to reflect a state of aberrant salience processing driven by elevated dopamine transmission (Kapur, 2003), and aberrant salience has been related to the presence of delusions in medicated patients with schizophrenia (Roiser *et al.* 2009). Similarly, individuals at ultra-high risk of psychosis demonstrate aberrant salience, the degree of which relates to the severity of delusion-like symptoms (Roiser *et al*. 2013). Since long-term, regular cannabis use is associated with increased risk of psychosis (Murray *et al.* in press), the aberrant salience hypothesis predicts that aberrant salience processing is elevated in this group and that this is linked to the induction of psychotic-like symptoms in cannabis users.

Therefore, we sought to investigate reward-based salience processing in cannabis users using the Salience Attribution Test (SAT) (Roiser *et al.* 2009). The SAT is a probabilistic reward-learning task featuring compound cue stimuli that vary along two dimensions, one task-relevant and one task-irrelevant. ‘Adaptive’ reward learning refers to differences in ratings (the explicit measure of learning) and reaction times (RTs) (the implicit measure of learning) along the task-relevant cue dimension, i.e. for high-probability reward cue features relative to low-probability reward cue features. ‘Aberrant’ reward learning is defined similarly, but along the task-irrelevant dimension, i.e. differences in ratings or RTs between cue features that are both associated with 50% probability of reward.

We hypothesized that cannabis users would show elevated levels of aberrant salience compared with non-user controls, and that within the cannabis users aberrant salience processing would be specifically associated with greater severity of transient psychotic phenomena. We also sought to explore whether users who meet diagnostic criteria for cannabis dependence or abuse [Diagnostic and Statistical Manual of Mental Disorders, fourth edition, text revision (DSM-IV-TR) 304.30; 305.20, i.e. compulsive or periodic harmful use of cannabis despite significant drug-related problems] would exhibit elevated aberrant salience processing. As a number of participants had previously undergone 3,4-dihydroxy-6-[^18^F]fluoro-l-phenylalanine ([^18^F]DOPA) positron emission tomography (PET) in this laboratory (Bloomfield *et al.* 2014*a*, *b*), we also sought to explore the relationships between dopamine synthesis capacity and aberrant salience processing.

## Method

### Study population

The study was approved by the National Research Ethics Service (Research Committee Reference 10/H0713/56) and conducted in accordance with the Declaration of Helsinki. All participants provided informed written consent to participate and received a modest financial reimbursement for their time.

Inclusion criteria for all participants were: minimum age of 18 years and capacity to give written informed consent. Exclusion criteria for all participants were: current or past psychiatric illness (excluding cannabis use disorders in the cannabis group) using the Structured Clinical Interview for DSM-IV (SCID) (First *et al.* 1996); family history of mental illness in a first-degree relative determined via the Family Interview for Genetic Studies (NIMH Genetics Initiative, 1992); evidence of an at-risk mental state for psychosis (Phillips *et al.* 2000); DSM-IV-TR (American Psychiatric Association, 2005) substance dependency or abuse (other than cannabis in the cannabis-user group and nicotine use disorders for all participants); and significant medical illness. None of the participants was taking psychotropic medication at the time of study participation.

Detailed drug histories were obtained from all participants using the Cannabis Experience Questionnaire (Barkus *et al.* 2006), structured interview and timeline follow-back (Sobell *et al.* 1996). Lifetime cannabis use was estimated as the total number of ‘spliffs’ (cannabis cigarettes; ‘joints’) consumed. The time taken to smoke an ‘eighth’ of cannabis (1/8 ounce; about 3.5 g, the standard unit of sale the UK) was chosen as the primary index of cannabis use because this provides a measure of the amount of current drug consumption (shorter time indicating greater consumption). This is likely to be more accurate than subjective recall of the number of spliffs consumed because of variability in cannabis dose between spliffs and inconsistencies in self-reported cannabis use (Akinci *et al.* 2001).

### Cannabis user group

All cannabis users were recruited by public advertisement. All participants were required to be current, at least weekly, users of cannabis. Cases were primarily recruited from an ongoing cohort study (Morgan *et al.* 2012). A subsample of users had measurements available on the induction of psychotic symptoms in response to smoking cannabis, which was defined as a positive change in scores on the psychotic items of the Psychotomimetic States Inventory (PSI) (Mason & Wakerley, 2012), measured 5 min after smoking their usual amount of cannabis (i.e. when acutely intoxicated) compared with when not intoxicated with the drug. These users consumed their own cannabis, and subjective ratings were acquired in the environment where users habitually consumed cannabis (e.g. at home) because drug effects are typically larger in naturalistic as opposed to laboratory environments (Barkus *et al.* 2006). Cannabis-induced psychotic symptoms abated within 2 h of consumption. The psychotic items from the PSI covered ‘delusional thinking’, ‘perceptual distortions’, ‘cognitive disorganization’ (thought disorder) and ‘paranoia’. Each item was rated on a four-point scale from ‘not at all’ (score = 0) to ‘strongly’ (score = 3). Examples of items include: ‘People can put thoughts into your mind’ and ‘You can sense an evil presence around you, even though you cannot see it’. A sample of the cannabis that each participant smoked was taken on the day of testing and analysed for levels of THC (Forensic Science Service, Birmingham, UK).

A total of 12 cannabis users who experienced a positive change in psychotic symptom severity in response to cannabis were recruited from the Bloomfield *et al.* (2014*a*, *b*) study. An additional two users were recruited from an ongoing study (Morgan *et al.* 2012). A further three users were recruited by public advertisement. Therefore 17, at least weekly, cannabis users are included in the present study. All cannabis users consumed the drug mixed with tobacco as a spliff.

### Control group

Non-user healthy control participants were recruited from the same geographical area by public advertisement. Controls were required to have no lifetime history of cannabis dependence or abuse (DSM-IV-TR), no more than 10 total uses of cannabis in their lifetime, no report of the induction of psychotic symptoms by cannabis, and no cannabis use in the preceding 3 months. Community surveys indicate that more than 30% of young adults in England report trying cannabis in their lifetime (Smith & Flatley, 2012). Control participants were therefore permitted to have had minimal exposure to cannabis to ensure that the control group was representative of the same general population from which the cannabis users were recruited.

### SAT

**Fig. 1.**
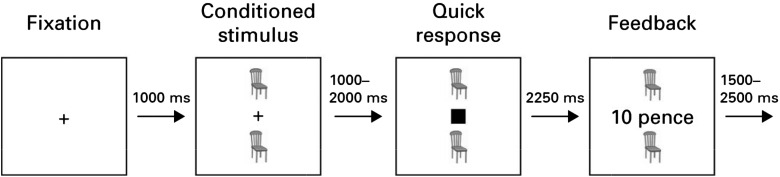
Salience Attribution Test. Subjects are presented with a fixation cross followed by a cue. They then have to respond to the solid square as quickly as possible. During 50% of trials, participants are rewarded with money for faster responses, with the probability of the reward signalled by the cue.

The SAT behaviourally measures aberrant salience. A more detailed description is provided in the original publication (Roiser *et al.* 2009) and the online Supplementary information. In brief, a cue stimulus appeared on the screen, which could vary across two dimensions: colour (red or blue) and form (animal or household object; Fig. 1). Stimulus features on one dimension predicted reward availability (e.g. red *v.* blue: 87.5 *v.* 12.5%); the other dimension was irrelevant in terms of reward occurrence (e.g. 50% reward for both animal and household features). Following the cue, participants had to respond to the presentation of a square (the probe) to win money. Faster responses yielded higher rewards, but reward was not always available. If the trial was not reinforced, the message ‘Sorry – no money available’ was displayed after the probe disappeared. If reinforced, ‘hit’ responses (made before the probe disappeared) that were slower than the participant's own mean RT (measured during an earlier practice session) resulted in the message ‘Hit – good: 10 pence’. For hit responses faster than the participant's mean practice RT the following messages appeared: ‘Quick – very good: X pence’ and ‘Very quick – excellent: X pence’. The maximum reward was £1. Participants performed the task in two separate blocks of equal length, over which values were averaged.

### PET

PET acquisition and analysis were performed as previously described (Bloomfield *et al.* 2014*a*, *b*) using a method that has demonstrated good test–retest reliability (Egerton *et al.* 2010). In brief, subjects underwent [^18^F]DOPA scanning on an ECAT HR+ 962 tomograph (CTI/Siemens, USA). Participants were asked to fast and abstain from cannabis for 12 h and to refrain from smoking tobacco for 2 h before imaging. On the day of PET scanning, urine drug screen (Monitect HC12; Branan Medical Corporation, USA) confirmed no recent drug use (other than cannabis in the user group), and a negative urinary pregnancy test was required in all female participants. A research clinician (M.A.P.B.) assessed psychotic symptoms using the Positive and Negative Syndrome Scale (PANSS) at the time of scanning. No participants had psychotic symptoms at the time of scanning [mean PANSS positive score cannabis users = 7.4 (s.d. = 0.5); control participants = 7.3 (s.d. = 0.5)]. Participants received carbidopa 150 mg and entacapone 400 mg orally 1 h before imaging to reduce the formation of radiolabelled [^18^F]DOPA metabolites (Cumming *et al.* 1993; Guttman *et al.* 1993). We performed a 10 min transmission scan before radiotracer injection for attenuation- and scatter-correction followed by bolus intravenous injection of approximately 180 MBq of [^18^F]DOPA. Emission data were acquired for 95 min over 26 frames. Head movement correction was performed with a wavelet filter (Turkheimer *et al.* 1999) and mutual information algorithm (Studholme *et al.* 1996). A summation image was created from each movement-corrected dynamic image using real-time position management (RPM) (Gunn *et al.* 1997). We then defined standardized regions of interest (ROIs) bilaterally in the whole striatum in Montreal Neurological Institute space (Martinez *et al.* 2003; Egerton *et al.* 2010) to create an ROI map. We used statistical parametric mapping software (SPM5; http://fil.ion.ucl.ac.uk/spm) to normalize the ROI map to each individual PET summation image using a template to aid normalization (Howes *et al.* 2009, 2011). We calculated the influx rate constant of [^18^F]DOPA uptake in each ROI relative to the cerebellum [*K*_*i*_^*cer*^ (min^−1^)] using the Patlak graphical analysis adapted for a reference tissue input function (Patlak & Blasberg, 1985; Hartvig *et al.* 1991, 1997; Hoshi *et al.* 1993).

### Statistical analysis

Data were analysed using the Statistical Package for the Social Sciences (SPSS), version 21 (IBM, USA). Demographic data were analysed using independent-samples *t* tests and χ^2^ tests. SAT data were analysed using repeated-measures analysis of variance with block (1/2) and probability as within-subject variables and group (cannabis user/control) as the between-subjects variable. Normality of distributions was assessed using the one-sample Kolmogorov–Smirnov test. Salience outcome measures were assessed for significant skew. RT and visual analogue scale (VAS) aberrant salience scores from the SAT were square root transformed prior to analysis to reduce skew, though untransformed values are presented in the text, figures and tables for clarity. Relationships between salience measures, symptoms and dopamine synthesis capacity were assessed using Pearson's *r*. To determine whether participants consistently assigned aberrant salience to any particular stimulus feature, χ^2^ tests were employed. For all analyses *p* < 0.05 (two-tailed) was considered significant.

## Results

### Participant characteristics

The mean age of first cannabis use was 15.5 (s.d. = 2.0) years, and the mean duration of at least weekly use was 5.9 (s.d. = 3.1) years. The mean time taken to smoke an eighth was 8.3 (s.d. = 7.3) days and mean lifetime exposure was 2850 (s.d. = 2447) spliffs. Six users met DSM-IV criteria for cannabis dependence or abuse. Mean time to smoke an eighth was 4.0 (s.d. = 4.3) days in users who met dependency and/or abuse criteria and 11.0 (s.d. = 8.4) days in users who did not meet criteria. A total of 17 control participants were matched to the user group for age (±5 years) and sex. Participant characteristics are reported in Table 1. Urine drug screens were positive for THC and negative for all other substances (amphetamine, opiates, cocaine, methamphetamine, benzodiazepines) in every cannabis user and negative for all drugs (including cannabis) in every control participant. There was no significant group difference in age or sex.

**Table 1.** Sample characteristics

	Controls (*n* = 17)	Cannabis users (*n* = 17)	*p* ^a^
Mean age, years (s.d.)	23.9 (4.2)	22.4 (1.9)	0.19
Sex, *n*			0.44
Female	6	3	
Male	11	14	
Mean cannabis use, g cannabis/month (s.d.)	n.a.	31.8 (38.5)	n.a.
Mean THC content of cannabis, % (s.d.)	n.a.	7.5 (2.9)	n.a.
Mean time to smoke an eighth of cannabis, days (s.d.)	n.a.	8.3 (7.3)	n.a.
Mean age of onset of regular cannabis use, years (s.d.)	n.a.	16.3 (2.0)	n.a.

s.d., Standard deviation; n.a., not applicable; THC, ∆^9^-tetrahydrocannabinol.

a Independent-samples *t* tests for variables with normal data distributions; Mann–Whitney *U* tests for variables with non-normal data distributions; χ^2^ tests for dichotomous variables.

### SAT

Behavioural data are presented in Table 2.

**Table 2.** Salience Attribution Test behavioural data

Test	Measure	Controls (*n* = 17)	Cannabis users (*n* = 17)
Block 1			
	RT high probability, ms	300.5 (114.9)	277.5 (111.7)
	RT low probability, ms	335.8 (51.4)	304.2 (53.2)
	RT adaptive salience, ms	11.2 (21.9)	3.8 (14.2)
	RT aberrant salience, ms	12.8 (4.7)	20.8 (19.5)
	VAS high probability, mm	55.8 (26.9)	63.0 (19.0)
	VAS low probability, mm	14.1 (8.4)	18.0 (12.1)
	VAS adaptive salience, mm	41.3 (29.4)	45.7 (25.3)
	VAS aberrant salience, mm	16.3 (14.5)	10.4 (9.6)
Block 2			
	RT high probability, ms	312.9 (56.4)	294.8 (57.5)
	RT low probability, ms	332.7 (58.1)	310.8 (67.0)
	RT adaptive salience, ms	20.3 (22.4)	14.9 (18.6)
	RT aberrant salience, ms	13.4 (15.2)	12.4 (7.7)
	VAS high probability, mm	63.3 (24.7)	66.3 (19.8)
	VAS low probability, mm	16.3 (9.7)	10.8 (7.6)
	VAS adaptive salience, mm	46.3 (26.7)	56.0 (23.1)
	VAS aberrant salience, mm	8.7 (6.4)	8.4 (8.6)
SPQ		–	19.9 (9.1)

Data are given as mean (standard deviation).

RT, Reaction time; VAS, visual analogue scale; SPQ; Schizotypal Personality Questionnaire.

### RT (implicit salience)

Participants responded faster on high- relative to low-probability-reinforced trials (*F*_1,31_ = 21.4, *p* < 0.001) and there was no group × probability interaction (*F*_1,30_ = 1.02, *p* = 0.32). There was no group × block interaction (*F*_1,32_ = 0.05, *p* = 0.82) and no main effect of group (*F*_1,32_ = 1.60, *p* = 0.22) or block (*F*_1,32_ = 2.43, *p* = 0.13). There was a significant probability × block interaction (*F*_1,32_ = 5.28, *p* = 0.03): across both groups implicit adaptive salience was significantly greater on block 2 than block 1.

There was no significant difference in implicit aberrant salience between cannabis users and controls (*F*_1,32_ = 1.12, *p* = 0.30), no group × block interaction (*F*_1,32_ = 1.08, *p* = 0.31) and no main effect of block (*F*_1,32_ = 1.30, *p* = 0.26). Participants did not consistently respond faster in the context of any particular irrelevant stimulus feature (*p* > 0.05).

### VAS (explicit salience)

Across all participants, high-probability-reinforced trials were rated as being more likely to yield reward compared with low-probability-reinforced trials (*F*_1,31_ = 130.0, *p* < 0.001). There was no main effect of block (*F*_1,32_ = 3.18, *p* = 0.08) and no group × block interaction (*F*_1,32_ = 0.38, *p* = 0.54). There was no significant effect of group on explicit adaptive salience (*F*_1,32_ = 0.80, *p* = 0.38, Fig. 3).

There was no significant effect of group on explicit aberrant salience (*F*_1,32_ = 1.09, *p* = 0.30) and no group × block interaction (*F*_1,32_ = 0.35, *p* = 0.56) or main effect of block (*F*_1,32_ = 2.43, *p* = 0.13). Participants did not consistently rate any particular irrelevant stimulus feature as more likely to yield reward relative to the others.

### Relationship between salience processing and cannabis use

Within the cannabis user group, there were no significant relationships between current cannabis use and measures of salience processing (implicit adaptive salience: *r* = 0.07, *p* = 0.79; implicit aberrant salience *r* = 0.49, *p* = 0.06; explicit adaptive salience *r* = −0.46, *p* = 0.07; explicit aberrant salience *r* = 0.14, *p* = 0.61). There was no significant relationship between age of onset of cannabis use and measures of salience processing (implicit adaptive salience: *r* = 0.32, *p* = 0.23; implicit aberrant salience *r* = −0.18, *p* = 0.52; explicit adaptive salience *r* = −0.12, *p* = 0.66; explicit aberrant salience *r* = −0.12, *p* = 0.65).

**Fig. 2.**
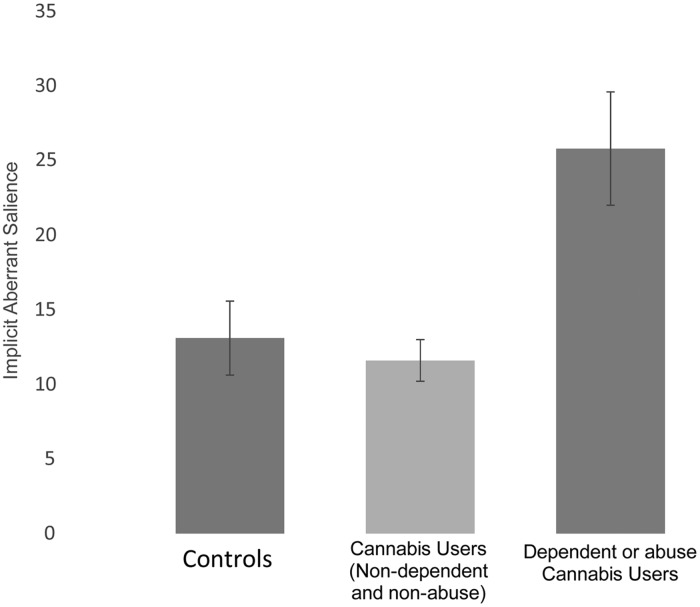
Implicit aberrant salience (ordinate; mm) based in controls and in cannabis users who meet Diagnostic and Statistical Manual of Mental Disorders, fourth edition (DSM-IV) dependency and abuse (*n* = 6), those who did not meet criteria (*n* = 11) and controls. Values are means, with vertical bars representing standard errors.

As an exploratory analysis, to examine whether cannabis dependency and abuse were associated effects of salience processing, the cannabis user group was divided into participants that met DSM-IV-TR criteria for cannabis dependency and/or abuse (*n* = 6) and those who did not meet criteria (*n* = 11). Within the cannabis users there was a significant effect of dependency and abuse diagnosis on implicit aberrant salience, with elevated implicit aberrant salience in the participants meeting dependent or abuse criteria relative to cannabis users not meeting these criteria [*F*_1,15_ = 5.8, *p* = 0.03, effect size (Cohen's *d*) = 1.97], but not on the other outcome measures (Fig. 2). However, when the control sample was included in the analysis, the effect of dependency or abuse diagnosis on implicit aberrant salience did not reach the threshold for statistical significance [*F*_2,32_ = 2.9, *p* = 0.07, effect size (Cohen's *d*) = 1.39].

### Relationship between aberrant salience processing and cannabis-induced psychotic symptoms

**Fig. 3.**
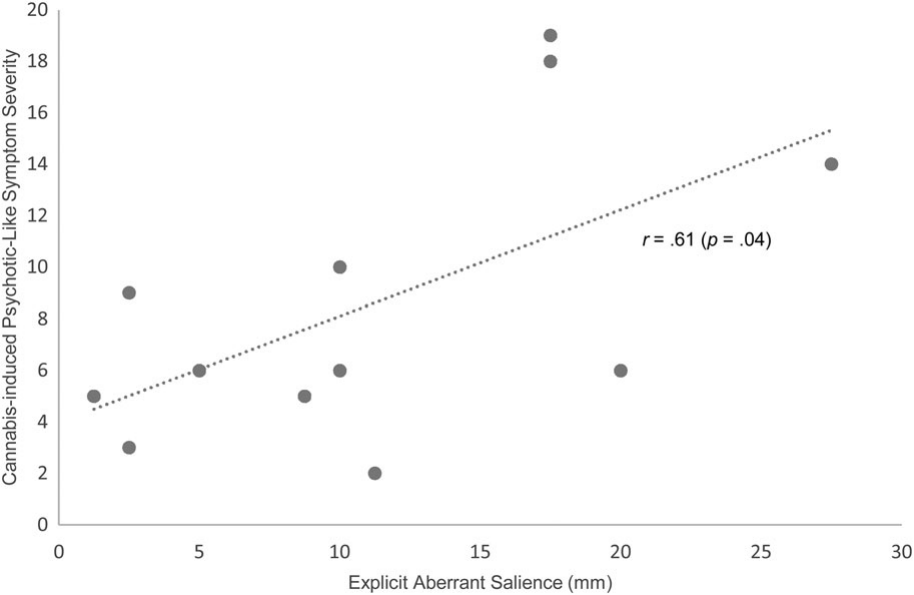
Relationship between explicit aberrant salience (mm) and cannabis-induced psychotic symptom severity (positive change in Psychotomimetic States Inventory Score).

Within the cannabis users, 12 experienced cannabis-induced psychotic symptoms [mean increase in PSI score = 8.6 (s.d. = 5.6)]. There was a significant relationship between cannabis-induced psychotic symptom severity and explicit aberrant salience (*r* = 0.61, *p* = 0.04; Fig. 3). There were no significant relationships between cannabis-induced psychotic symptoms and the other salience measures (*p* > 0.05), or between Schizotypal Personality Questionnaire score and salience measures (*p* > 0.05).

### Relationship between salience processing and dopaminergic function

**Fig. 4.**
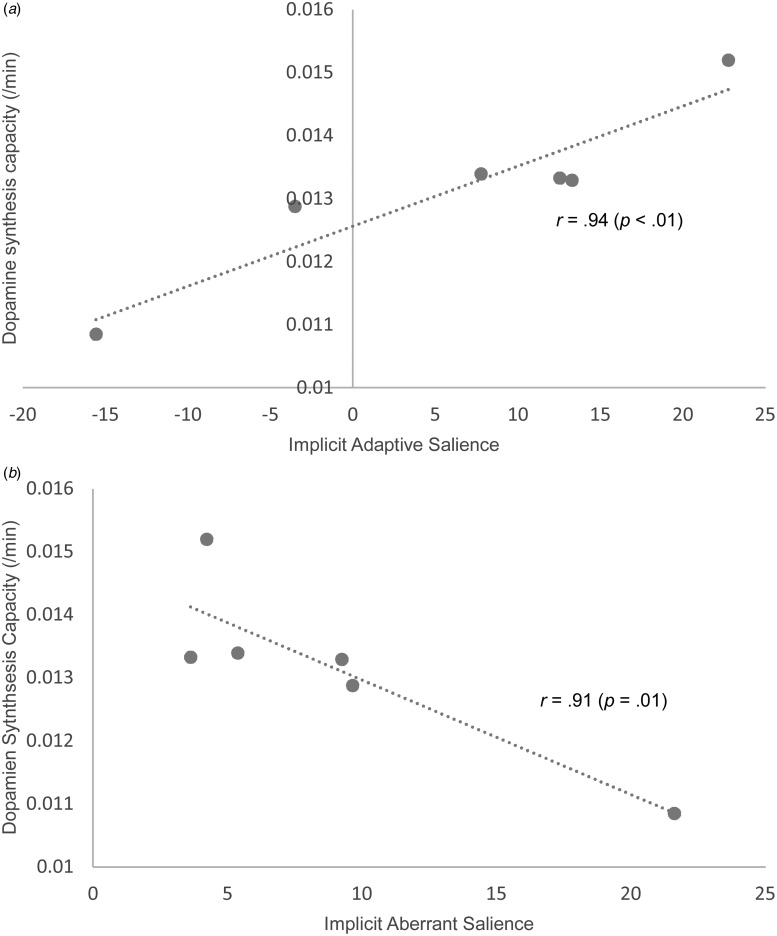
Relationships between dopamine synthesis capacity (indexed as the influx rate constant *K*_*i*_^*cer*^) in the whole striatum and implicit adaptive salience (*a*) and implicit aberrant salience (*b*) in controls.

As an exploratory analysis, data are presented on salience processing and dopaminergic function. Six controls in the present study had participated in the study of dopaminergic function in cannabis users (Bloomfield *et al.* 2014*a*, *b*). Both implicit and explicit adaptive salience was positively correlated with whole striatal dopamine synthesis capacity, whilst implicit aberrant salience was inversely correlated with whole striatal dopamine synthesis capacity (Fig. 4; Table 3).

**Table 3.** Relationships between salience attribution and dopamine synthesis capacity (indexed as *K_i_^cer^*) in the striatum in controls who had previously undergone PET scans (*n* = 6)

*K*_*i*_^*cer*^, min^−1^		RT adaptive salience		RT aberrant salience		VAS adaptive salience		VAS aberrant salience	
Mean	(s.d.)	*r*	*p*	*r*	*p*	*r*	*p*	*r*	*p*
0.0132	(0.0014)	0.94	0.006	−0.91	0.01	0.82	0.05	−0.15	0.78

*K*_*i*_^*cer*^, Influx rate constant; PET, positron emission tomography; RT, reaction time; VAS, visual analogue scale; s.d., standard deviation.

Of the cannabis users in the present study, 10 had participated in our previous study of dopaminergic function in cannabis users. There were no significant relationships between the SAT outcome measures and dopamine synthesis capacity in the whole striatum (Table 4). Fisher's *r*-to-*z* transformation was applied to examine whether differences in the relationships between dopaminergic functioning and salience processing between users and controls were significant (Table 5). Significant differences were found in the relationships between both implicit adaptive and aberrant salience processing and dopamine synthesis capacity in the whole striatum. Specifically, cannabis use was associated with the loss of a positive relationship between implicit adaptive salience and dopamine synthesis capacity, and the loss of an inverse relationship between implicit aberrant salience and dopamine synthesis capacity.

**Table 4.** Relationships between salience attribution and dopamine synthesis capacity (indexed as *K_i_^cer^*) in the striatum in cannabis users who had previously undergone PET scans (*n* = 10)

*K*_*i*_^*cer*^, min^−1^		RT adaptive salience		RT aberrant salience		VAS adaptive salience		VAS aberrant salience	
Mean	(s.d.)	*r*	*p*	*r*	*p*	*r*	*p*	*r*	*p*
0.0128	(0.0008)	0.27	0.45	−0.11	0.77	0.55	0.10	0.22	0.55

*K*_*i*_^*cer*^, Influx rate constant; PET, positron emission tomography; RT, reaction time; VAS, visual analogue scale; s.d., standard deviation.

**Table 5.** Fisher's *r*-to-*z* transformation to examine significant differences in the relationships between salience processing and striatal dopamine synthesis capacity in cannabis users and controls

	RT adaptive salience		RT aberrant salience		VAS adaptive salience		VAS aberrant salience	
ROI	*z*	*p*	*z*	*p*	*z*	*p*	*z*	*p*
Striatum	2.12	0.03	−2.05	0.04	0.78	0.44	−0.54	0.59

RT, Reaction time; VAS, visual analogue scale; ROI, region of interest.

## Discussion

The main finding from this study is that within cannabis users who experienced cannabis-induced psychotic symptoms, there was a significant relationship between cannabis-induced psychotic symptom severity and aberrant salience processing, accounting for 37% of the variance in psychotic symptom severity. Whilst regular long-term cannabis use was not associated with statistically significant differences in behavioural measurements of salience processing, which is inconsistent with our primary hypothesis, these results show preliminary evidence of increased aberrant salience in cannabis users who meet DSM-IV criteria for cannabis abuse or dependence (effect size: Cohen's *d* = 1.2), suggesting that aberrant salience may only become apparent when there is cannabis dependence. In an exploratory analysis, within controls there were positive relationships between both measures of adaptive salience and whole striatal dopamine synthesis capacity, whilst there was an inverse relationship between implicit aberrant salience and whole striatal dopamine synthesis capacity. However, no significant relationships between whole striatal dopamine synthesis capacity and salience processing were observed in cannabis users. The results also indicate a loss of relationship between implicit salience processing and dopamine synthesis capacity in the whole striatum associated with long-term cannabis use.

This is the first study to examine aberrant salience processing in cannabis users. Whilst there was no significant difference in aberrant salience between the cannabis users and controls, a finding of increased implicit aberrant salience in cannabis users who meet DSM-IV-TR criteria for abuse or dependence compared with those who do not suggests that cannabis dependence and abuse are associated with increased aberrant salience. We also found that cannabis-induced psychotic symptom severity and explicit aberrant salience are significantly positively correlated, in line with findings of a positive relationship between explicit aberrant salience and delusion-like symptoms in people at ultra-high risk of psychosis (Roiser *et al*. 2013) and delusional symptoms in people with schizophrenia (Roiser *et al.* 2009). In addition, there were some novel findings not predicted by the aberrant salience hypothesis. These were that in healthy controls, whole striatal dopamine synthesis capacity was positively correlated with both measures of adaptive salience processing and negatively correlated with implicit aberrant salience. The finding of opposite relationships between dopamine synthesis capacity and salience processing in healthy controls is not predicted by the aberrant salience hypothesis, where increased dopamine synthesis capacity is predicted to be related to increased aberrant salience and not vice versa. Two studies have assessed previously assessed dopamine synthesis and aberrant salience. One of these did not find significant relationships between the measures (Roiser *et al*. 2013) and a more recent, larger study, reported a positive relationship between right ventral striatal dopamine synthesis capacity and aberrant salience (Boehme *et al.* 2015). However, the former study did report that higher dopamine synthesis capacity predicted greater adaptive reward prediction haemodynamic responses in controls, whereas the opposite relationship applied in the individuals at ultra-high risk of psychosis, in line with the findings in control participants in the current study. Roiser *et al.* (2013) speculated that the positive impact of high dopamine synthesis capacity on motivational salience signalling may depend on the baseline state of the dopamine system, such that in healthy volunteers, high dopamine synthesis capacity may facilitate the transmission of motivational salience, potentiating appropriate phasic signals against a background of relatively low gain or tonic dopamine release. Taken together with findings that there is a loss of relationship between implicit salience processing and dopamine synthesis capacity in the whole striatum associated with long-term cannabis use, and given that the mesolimbic dopamine system plays a central role in normal salience processing (Zink *et al.* 2003) which is modulated by endocannabinoid signalling (Fernandez-Ruiz *et al.* 2010; Melis & Pistis, 2012; Melis *et al.* 2012), this would suggest that long-term cannabis use may give rise to aberrant salience by disrupting dopaminergic salience processing. Alternatively, this may predate the cannabis use, such that these individuals then experience a greater reward from smoking cannabis. Whilst the effects of acute THC on aberrant salience processing using the SAT have yet to be reported in the literature, and the case–control design of this study is not able to infer causality, there is evidence from a study using the oddball task (Bhattacharyya *et al.* 2012) that THC reduces latency to non-salient *v.* salient stimuli in healthy volunteers, consistent with this interpretation. However, this phenomenon may not be restricted to reward-based learning only, as increased speed and error rates were observed with THC challenge in a learning and episodic memory task (Curran *et al.* 2002). Nonetheless, long-term cannabis use has been associated with impairments in filtering out non-salient information during a selective attention task (Solowij *et al.* 1991) and THC resulted in irrelevant background visual and auditory stimuli becoming more salient during the performance of a visual processing task (D'Souza *et al.* 2004).

Adolescence is a period of vulnerability to the development of neurocognitive effects associated with cannabis use and there is also growing evidence that cannabis use is associated with multiple cognitive endophenotypes that are in common with schizophrenia such as response inhibition, sustained attention, working memory and executive function (Solowij & Michie, 2007). Yet, behavioural studies have demonstrated that acute THC challenge produces transient, acute psychotic reactions, the extent of which are unrelated to the degree of cognitive impairment or anxiety. There is a large body of evidence describing the vulnerability of adolescents to impaired cognition, across a range of domains, associated with cannabis use (Jager & Ramsey, 2008). Animal studies indicate that brain CB_1_ receptor levels peak in early adolescence (Belue *et al.* 1995) and humans exposed to cannabis in adolescence are more likely to have impaired neurocognitive function than individuals exposed in adult life (Fontes *et al.* 2011). Furthermore, there is evidence that neurocognitive deficits (such as impaired RTs, attention and memory) associated with adolescent cannabis use can persist after abstinence (Medina *et al.* 2007). As described by Schmidt & Roisier (2009) in order to perform the SAT, participants must be able to attend continuously for an extended period, use working memory, learn probabilistic associations and guide responses on the basis of such associations, all of which may be impaired with cannabis use (Pope *et al.* 2001; Scholes & Martin-Iverson, 2009). In order to examine whether other cognitive processes (including working memory, sustained attention, probabilistic reversal learning) were influencing measures on the SAT, Schmidt & Roisier (2009) performed a factor analysis using the SAT with a battery of cognitive tasks. They found that the SAT could dissociate aberrant salience processing from other aspects of reward learning and attention, although adaptive salience and learned irrelevance were associated with each other. It is therefore unlikely that other aspects of cognitive function that are affected by cannabis use are influencing the current results, although these were not verified in the current study. However, the cannabis users in this study had faster RTs than non-users on both high- and low-probability items in both blocks of the SAT, suggesting that generalized psychomotor slowing in cannabis users is unlikely to account for the current results.

A potential limitation of the current study is that participants consumed their own cannabis, rather than a standard preparation. However, individuals were tested whilst intoxicated and the levels of THC in samples of the cannabis participants were using were measured and it was confirmed that the cannabis contained high levels of THC. There was no fixed interval between cannabis exposure and SAT session, meaning that heavier cannabis users may have had a shorter interval between exposure and scan. It therefore remains possible that differences in the time since last cannabis exposure, and therefore acute *v.* chronic effects of cannabis, contribute to the differences between the dependent/abuser and non-dependent groups, rather than dependency and/or abuse *per se*. The measures of substance use rely on self-report and it was not possible to independently verify substance use histories beyond ongoing cannabis use in the user group and no recent use of other drugs in all participants.

A recently published study (Bianconi *et al.* 2016) found differences in cannabis-related experiences between patients with a first episode of psychosis and controls. The authors of that study reported that patients with a first episode of psychosis exhibit a hypersensitivity to cannabis which not only involved frequent ‘unpleasant experiences’ but also increased ‘enjoyable feelings’. The authors hypothesized that that the increased positive reward acted as a reinforcer to increase the risk of developing cannabis dependence and counterbalancing the experience of unpleasant effects. A large randomized, placebo-controlled study found that THC increased paranoia by increasing negative affect (i.e. anxiety) (Freeman *et al.* 2014). A further limitation of this study would therefore be that measures of anxiety, such as the Beck Anxiety Inventory, were not recorded. However, taken together with the current study, this suggests that heavy cannabis use may result in a combination of aberrant salience, anxiety, paranoia and amotivation (Bloomfield *et al.* 2014*b*), which might explain the increased risk of schizophreniform psychosis. Future work should therefore assess the relationships between both long-term cannabis use and acute THC on psychotic symptoms, salience processing, paranoia, amotivation and negative affect in order to examine this hypothesis.

## Conclusion

These results suggest that cannabis dependence and abuse are associated with increased aberrant salience processing, and that within cannabis users there is a positive relationship between explicit aberrant salience and cannabis-induced psychotic symptom severity. There is also evidence that long-term cannabis use is associated with altered relationships between striatal dopamine synthesis capacity and salience processing. Long-term cannabis use may therefore increase the risk of psychotic symptoms by increasing aberrant salience via disrupted striatal dopaminergic processing.

## Supplementary material

For supplementary material accompanying this paper visit http://dx.doi.org/10.1017/S0033291716002051. click here to view supplementary material
