# Supplementary material for: Salience attribution and its relationship to cannabis-induced psychotic symptoms
Source: Psychol Med. 2016 Sep 15;46(16):3383–95. doi: 10.1017/S0033291716002051 (PMC5122315; doi:10.1017/S0033291716002051)
Supplement: Supplementary file 1 [file S0033291716002051sup001.doc]

**Supplementary information**

During the SAT (1), participants made a speeded response to the onset of a probe (a black square) in order to earn money. Cues (pictures) appearing immediately before the onset of the probe signaled the probability that the participant would win money on a given trial, which occurred on 50% of trials. However, participants were not informed of the contingencies between the different pictures and reward. Participants could earn a maximum of £20 on the test (minimum £5). Participants completed a computerized tutorial, featuring example displays, written instructions and test trials before the main test. The tutorial contained two practice sessions, without cues or money, to familiarize participants with the test and provide a measure of baseline response time (RT). During practice sessions, a fixation cross appeared at the beginning of each trial. Following a variable interval (minimum 0.5 s, maximum 1.5 s) the probe appeared, and participants responded by pressing a button as quickly as possible. Participants were instructed to try to respond as quickly as they were able to, and before the box disappeared. During the first practice session the probe was on the screen for randomized variable periods, with a maximum duration of 1.5 s, minimum duration 0.5 s and mean duration 1 s. Feedback was provided after 2 s as ‘Good’ if the participant responded before the box disappeared, ‘Try to respond faster’ if they responded after the box disappeared, ‘Too early’ if they responded before the box appeared, and ‘No key pressed’ if they made no response. On the second practice session, the mean probe duration was set to be the mean RT from the first, ensuring participants were responding as quickly as possible and to yoke task difficulty to individual performance. The standard deviation (SD) of the fastest half of the trials (SDF) was also calculated, and was used to set the minimum and maximum probe durations for the second practice session (mean from first practice session±2×SD). For the main test, the mean, minimum and maximum probe durations were calculated from the second practice session in the same way.

Participants then completed two blocks of 64 trials on the main test, where money was available on 50% of trials. The likelihood that money was available on a trial was signaled by one of four cues that appeared at the top and bottom of the screen before the onset of the probe. Cues varied on two different visual dimensions: color (blue or red) and shape (animal or household object). Therefore, there were four different types of cues: blue animals; red animals; blue household objects and red household objects. One of these dimensions (e.g. color) was task-relevant so that one level of the dimension was reinforced on 28 out of 32 (87.5%) of the trials while only four out of 32 (12.5%) trials of the other were reinforced. For example, if ‘color’ was the reinforced dimensions, 14 out of 16 blue animals and 14 out of 16 blue household objects would be reinforced, compared with only two out of 16 red animals and two out of 16 red household objects. The other dimension, in this example ‘shape’, was task-irrelevant, so that 16 out of 32 (50%) of both levels were reinforced. The contingencies were identical on the first and second blocks of the game. Participants were not informed of these contingencies, but instead learned them over the course of the game.

Four different versions of the SAT were used, each with a different stimulus feature (blue, red, animal or household object) reinforced with high probability. Each participant was administered the same version for both blocks of the SAT.

If the trial was not reinforced, the message ‘Sorry – no money available’ was displayed. If the trial was reinforced, participants won between 5 and 100 pence, depending on the latency of their response. On reinforced trials where participants either made no response or responded after the probe had disappeared, the message ‘Missed: 5 pence’ was displayed. If participants responded prematurely (<100 mms after the onset of the probe), the message displayed was ‘Too early: 5 pence’. On reinforced trials where participants responded before the probe disappeared, but slower than their mean RT, the message ‘Hit – good: 10 pence’ was displayed. When participants responded more quickly than their mean RT, the message ‘Quick – very good: *X* pence’ was displayed (for responses up to 1.5 SDs faster than their mean RT) and ‘Very quick – excellent: *X* pence’ (for responses faster than their mean RT by at least 1.5 SDs). The reward was scaled according to *X*=10+90×(mean RT – trial RT)/(3×SD), up to a maximum of 100 pence. For example, a response 1 SD faster than the mean was reinforced with 40 pence, a response 2 SDs faster was reinforced with 70 pence, and any responses 3 SDs or faster than the mean were reinforced with 100 pence. The money won on each trial was added to the participant's running total for that block, *Y*, which was displayed underneath the feedback: ‘Total – £*Y*’. On reinforced trials, a 0.5 s tone sounded, frequency: (300+(10×X)) Hz. At the end of each block, participants indicated, using 10 mm visual analogue scales (VAS), their estimate of the reinforcement probabilities for each of the four different cues (see figure 1).

Two types of motivational salience were calculated for each block: “adaptive” and “aberrant”. Adaptive salience was defined in two ways: behaviorally (implicit measure) and subjectively (explicit measure). RT adaptive salience (implicit) was defined as the speeding of responses on high-probability-reinforcement trials relative to low-probability-reinforcement trials (collapsing across the task-irrelevant stimulus dimension), and VAS adaptive salience (explicit) was defined as the increase in probability rating for high-probability-reinforcement trials relative to low-probability reinforcement trials (again, collapsing across the task-irrelevant stimulus dimension). Aberrant salience was defined as the absolute difference in RT (implicit) or VAS rating (explicit) between the two levels of the task-irrelevant stimulus dimension (collapsing across the task-relevant stimulus dimension). Since aberrant salience is defined as any deviation from equal reaction time or subjective reinforcement probability rating for the two levels of the task-irrelevant stimulus dimension, the sign is unimportant. Therefore aberrant salience was always positive, whereas adaptive salience could be positive or negative. The number of premature responses and omissions were also recorded for each stimulus type on each block.

**Sample size**

Explicit aberrant salience was the primary outcome measure and the study was powered for this variable. Explicit aberrant salience was the primary outcome measure and the study was powered for this variable.  In a previous study, the correlation between aberrant salience and psychotic symptoms in individuals at ultra-high risk of psychosis was *r* = .62 (2).  Using G*Power software, we performed an a priori power calculation and found that in order to achieve a power of .8 with an expected relationship between cannabis-induced psychotic symptoms and aberrant salience of *r* = .62, alpha set at *p*<.05 (two-tailed), it was calculated that at least 15 participants would be required in the cannabis user group.

**References**

1. Roiser JP, Stephan KE, den Ouden HE, Barnes TR, Friston KJ, Joyce EM (2009) Do patients with schizophrenia exhibit aberrant salience? *Psychol Med.* 39(2):199-209.
2. Roiser JP, Howes OD, Chaddock CA, Joyce EM, McGuire P (2013) Neural and behavioral correlates of aberrant salience in individuals at risk for psychosis. *Schizophr Bull.* 39(6):1328-36.
